# Passive Smoking and Oral Health of Infants, Preschoolers, and Children: A Systematic Review

**DOI:** 10.1093/ntr/ntad093

**Published:** 2023-06-13

**Authors:** Thusheka Uthayakumar, Josephine Xanthe Bennett, Hazel Leah Cartas, Mylène Brunet, Kim Loan Vo, Jeroen Kroon

**Affiliations:** School of Medicine and Dentistry, Griffith University, Gold Coast, Queensland, Australia; School of Medicine and Dentistry, Griffith University, Gold Coast, Queensland, Australia; School of Medicine and Dentistry, Griffith University, Gold Coast, Queensland, Australia; School of Medicine and Dentistry, Griffith University, Gold Coast, Queensland, Australia; School of Medicine and Dentistry, Griffith University, Gold Coast, Queensland, Australia; School of Medicine and Dentistry, Griffith University, Gold Coast, Queensland, Australia

## Abstract

**Introduction:**

Almost half of the world’s children experience passive smoking, which is linked to numerous oral health conditions. The aim is to synthesize data on the impact of passive smoking on oral health of infants, preschoolers, and children.

**Aims and Methods:**

A search was conducted across Medline (via EBSCOhost), PubMed, and Scopus up to February 2023. Risk of bias was assessed according to the Newcastle-Ottawa Scale (NOS).

**Results:**

The initial search produced 1221 records and after removal of duplicates, screening by title and abstract, and full-text assessment, 25 studies were eligible for review and data extraction. The majority of studies (94.4%) found a correlation between passive smoking and increased prevalence of dental caries with three studies suggesting a dose–response relationship. Prenatal passive smoking exposure in 81.8% of studies indicated an increased dental caries experience compared to postnatal exposure. Low parental education, socioeconomic status, dietary habits, oral hygiene, and gender affected the level of environmental tobacco smoke (ETS) exposure and dental caries risk.

**Conclusions:**

The results of this systematic review strongly suggest a significant association between dental caries in the deciduous dentition and passive smoking. Early intervention and education on the effects of passive smoking on infants and children will allow for the improvement in oral health outcomes and reduction in smoking-associated systemic conditions. The results justify all health professionals paying more attention to passive smoking when conducting pediatric patient histories, contributing to improved diagnosis and appropriate treatment planning with more suitable follow-up schedules.

**Implications:**

The evidence from this review that environmental tobacco smoke and passive smoking is a risk factor for oral health conditions, both prenatally and postnatally during early childhood, justifies all health professionals paying more attention to passive smoking when conducting pediatric patient histories. Early intervention and appropriate parental education regarding the effects of secondhand smoke on infants and children will allow for the minimization of dental caries, improvement in oral health outcomes and overall reduction in smoking-associated systemic conditions for the children exposed.

## Introduction

Globally tobacco smoking is one of the greatest burdens on public health, affecting the risk of numerous health conditions, and leading to more than 8 million deaths each year.^1^ Passive smoking occurs when you breathe in environmental tobacco smoke (ETS), also known as secondhand smoke (SHS). This contributes to over 15% of the mortality rate attributed to tobacco.^1^ It is estimated that almost half of the world’s children are exposed to passive smoking with the family home as the main source.^2^ Over 4000 chemicals and carcinogens have been isolated from ETS, which have been shown to increase the risk of multiple organ cancers, heart and lung disease, stroke, metabolic, and respiratory conditions.^3^ Prenatal passive smoking has been linked to preterm delivery, low birth weight, sudden infant death syndrome, developmental delays, and congenital defects such as cleft lip or palate.^4^

Cotinine, the primary metabolite of nicotine, will only be present in the body when exposed to tobacco smoke. The half-life of cotinine ranges from 16 to 19 h, significantly longer than that of nicotine, and it can be measured in blood, urine, or saliva. Cotinine is thus considered to be a reliable biomarker to measure exposure to ETS and passive smoking.^5^

Passive smoking has been linked to numerous oral health conditions, ranging from halitosis, staining, reduced taste, smoker’s keratosis, leukoplakia, and oral cancers.^6^ The association between passive smoking and gingival pigmentation, periodontal disease, and dental caries has been extensively investigated in older children and adults. Stimulated melanocyte production in the oral epithelium, causing dark gingival pigmentation and melanosis in adults and children aged 10–11, has been attributed to ETS and passive smoking.^7^ Children aged 6–12 had a reduction in periodontal attachment when exposed to passive smoking. Multiple cross-sectional studies have suggested a link between pre- or postnatal passive smoking and an increased dental caries risk.^8–14^ Despite these significant associations in older age groups, no systematic review on the relationship between passive smoking and oral conditions in younger children aged 0–6 has been published. This population is considered potentially more vulnerable, with immature immune systems, lower salivary flow, rapidly developing oral structures, the deciduous dentition more susceptible to hypoplastic defects, thinner enamel, increased risk of gingivitis, and potentially extended durations of passive smoking at home.^8,15,16^

The aim of this systematic review was to investigate the association between passive smoking and oral health in the deciduous dentition of infants, preschoolers, and children from birth to age 6.

## Methods

The systematic review was conducted following Preferred Reporting Items for Systematic Reviews and Meta-Analysis (PRISMA) guidelines.^17^ The protocol for this review was also registered on PROSPERO (CRD42022297437).

### Eligibility Criteria

The Population–Exposure/Event–Comparison–Outcome (PECO) framework guided the controlled terminology (Medical Subject Headings [MeSH] terms) and keywords used in the search strategy.^18^ Population: 0- to 6-year-old infants, preschoolers, and young children; exposure/event: ETS and passive smoking; comparison/control: not applicable to this review; and outcome: oral health conditions in the deciduous dentition.

Eligibility criteria included: (1) observational studies (cohort, case–control, and cross-sectional); (2) peer-reviewed journals; (3) published in English; (3) 0- to 6-year-olds (defined by MeSH as either infants, preschoolers, and young children); (4) deciduous dentition; (5) pre- and/or and postnatal smoking; and (6) whether exposure was maternal or household.

Exclusion criteria included: (1) case reports, case series, pilot study, letters/editorials, opinion-based studies; (2) children older than six; (3) research topic focus excludes oral health; (4) non-English text; (5) no conclusions drawn; (6) secondary data; (7) full text unavailable; and (8) articles published before 2000.

Where studies presented results for both the deciduous and permanent dentitions in multiple age groups, the study was only included if the data were presented separately for the deciduous dentition and 0- to 6-year-old children.

### Search Strategy

An electronic search was undertaken in February 2022 in Medline (via EBSCOhost), PubMed, and Scopus. Bibliographies of included articles were scanned manually to identify any additional relevant articles. The search was repeated in February 2023 to ensure inclusion of any newly published articles. The search strategy is shown in Table 1. Titles and abstracts were independently screened by two reviewers (MB, TU). The level of agreement was calculated using Cohen’s kappa and was set at 0.85.^19^ A third reviewer (XB) resolved any discrepancies and disagreement. Following removal of duplicates, the full-text analysis was independently performed by three reviewers (XB, MB, TU). Reasons for exclusion were recorded.

**Table 1.** Search Strategy

#	Search term
#1	Dental caries OR caries OR tooth decay [all fields]
#2	Oral health [all fields]
#3	Periodontal disease OR gingivitis OR gingival pigmentation OR bleeding on probing OR probing depth OR clinical attachment level OR loss of attachment OR plaque ind*[all fields]
#4	Salivary cotinine [all fields]
#5	(#1 OR #2 OR #3 OR #4)
#6	Children OR child*[all fields]
#7	Infant* OR baby OR babies OR newborn* [all fields]
#8	Neonate* [all fields]
#9	(#6 OR #7 OR #8)
#10	Tobacco OR nicotine [all fields]
#11	Passive smok* OR second hand smok* OR household smok* OR involuntary smok* OR environmental smok* OR environmental tobacco* [all fields]
#12	(#10 OR #11)
#13	#5 AND #9 AND #12

### Risk of Bias

Risk of bias was assessed according to the Newcastle-Ottawa Scale (NOS) for cohort and case–control studies,^20^ and a modified version of this tool for cross-sectional studies.^21^ This instrument evaluates selection, exposure, and comparability with a maximum score of nine for cohort and case–control studies, and a maximum score of 10 for cross-sectional studies. A score equal to or less than five indicates a high risk of bias, six to seven a medium risk, and eight or higher a low risk of bias. The scores for each study were independently rated and compared by two reviewers (HC, KV). Disagreement was resolved by consensus.

### Data Extraction and Analysis

Data extraction was independently done by three reviewers (XB, MB, TU) using the Cochrane Checklist of items.^22^ This included study type and details (author, year and publication, and country), sample size, and age of participants. Health variables (including dental caries, gingivitis, gingival pigmentation, and presence of salivary cotinine), as well as smoking exposure (pre-/postnatal and maternal/household), were recorded. Confounding factors identified for each included paper were also recorded. These included parental education, family income, diet, fluoride exposure, gender, and oral hygiene practices. This allowed for more reliable conclusions whether passive smoking had a direct effect on oral health, or if the results were affected by these additional confounding factors.

### Frequency Effect Size

The frequency effect size (FES) for health variables and smoking exposure was calculated by dividing the number of studies reporting either a positive or negative association by the total number of included studies assessing this factor, expressed as a percentage.

## Results

### Study Selection and Characteristics

**Figure 1.**
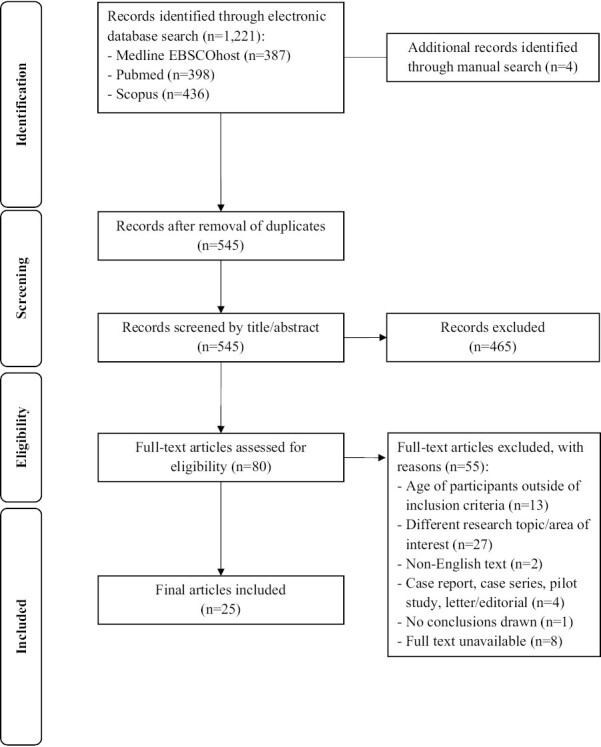
PRISMA flow chart.

The PRISMA flowchart for the final selection of studies is depicted in Figure 1. The electronic literature search of the included databases produced 1221 records. A manual search identified four additional records. After removal of duplicates, the remaining 545 records were screened by title and abstract for relevance. This left 80 records for full-text assessment. A further 55 records were excluded based on the predefined inclusion and exclusion criteria. This resulted in 25 studies eligible for review and data extraction.

### Descriptive Analysis

A summary of the 25 included articles and their extracted data is presented in Supplementary Table S1. Table 2 presents FES. The majority of included studies were cross-sectional (*n* = 19), five were cohort studies, and the remaining study one was a case–control. Studies were conducted in 12 different countries, nine of which in Japan. Dental caries was assessed by dental practitioners, and passive smoking was evaluated by salivary cotinine.

**Table 2.** Frequency Effect Size (FES) of Health Variables and Smoking Exposure

	Studies assessing the risk factor (*n*)	Positive association with passive smoking *n* (%)	No association with passive smoking *n* (%)
Health variables			
Dental caries	18	17 (94.4)	1(5.6)
Salivary cotinine	7	7 (100)	0 (0)
Smoking exposure			
Prenatal	11	9 (81.8)	2 (18.2)
Postnatal	20	19 (95)	1 (5)
Maternal	12	12 (100)	0 (0)
Household	21	19 (90.5)	2 (9.5)

### Risk of Bias

Risk of bias for all studies is presented in Supplementary Table S2. Of the five cohort studies, one was assessed as low, while the remaining four were identified as a high risk of bias. The single case–control study had a high risk of bias. The majority of cross-sectional studies (*n* = 12) had a low, five a medium, and two a high risk of bias.

### Data Synthesis

#### Dental Caries

Of the included studies, 18 investigated the association between passive smoking and the prevalence of dental caries (Supplementary Table S1). Of these, 94.4% (*n* = 17) found that passive smoking was independently associated with the prevalence of dental caries.^11,12,14,16,23–35^ The remaining study, in a univariate analysis, found that passive smoking was significantly associated with the development of dental caries, however, after adjusting for risk factors, passive smoking was no longer an independent risk factor.^36^

Odds ratios were often described to demonstrate the effect of exposure versus non-exposure; this ranged from OR 1.33 to 3.14.^34^ A threefold increase for childhood dental caries in children exposed to maternal smoking was reported in one study, and if the mother reported smoking of one pack a day for 3 years; this risk increased by fivefold.^27^ Boys were found to have an increased dental caries prevalence compared to girls after adjusting for covariates in one study.^11^

Dental caries was reported using different indices. The decayed, missing, and filled teeth (dmft) or surfaces (dmfs) indices were the most common, with a score of one or higher signifying dental caries. Only one study reported the use of radiographs to confirm the presence of dental caries, although these were not available for all subjects.^29^ A few studies reported that radiographs were unnecessary in young children and that it was unethical to expose children to radiation for the purposes of research.

Parental smoking was not significantly associated with dental caries in 3-year-olds after adjusting for risk factors in four studies.^23,29,33,34^ However, the association remained significant in the 5-year-old group, which may be explained by the cumulative effect of exposure of SHS over the additional years, and the opportunity for new dental caries formation.

A dose–response relationship was reported in three studies, where the greater the number of cigarettes smoked correlated to greater severity in childhood dental caries.^24,27,34^

#### Salivary Cotinine

Salivary cotinine was investigated in one cohort and six cross-sectional studies, all of which showed a positive association with passive smoking.^5,37–42^ In all of these saliva samples were centrifuged and frozen until analysis. These results confirm that passive smoking has a direct effect on the salivary cotinine level. Salivary cotinine was higher where parents smoked more cigarettes per day, or if there were no household smoking restrictions, such as where household members were smoking indoors or on the balcony compared to outdoors.^39^ Lower socioeconomic status (SES) households implement fewer smoking restrictions, again confirming that more direct passive smoking leads to higher salivary cotinine levels in children.^41^ The specific level of particulate matter in the air of a smoking household, as a means of determining the degree of passive smoking in the environment, was measured in one study.^38^ This is considered to be a more accurate method of determining the degree of passive smoking compared to surveys alone. The positive association between salivary cotinine levels and passive smoking, as well as a dose–response relationship, was reported by this study.^38^

Salivary cotinine levels in newborns and their mothers and fathers smoking indoors were significantly higher compared with non-smoking and outdoor smoking fathers.^39^

#### Gingivitis and Gingival Pigmentation

Only one cross-sectional study evaluated the association between passive smoking and gingival pigmentation as well as gingivitis using the Loe and Silness Gingival Index.^28^ Children exposed to passive smoking showed no association with gingivitis. Additionally, children not exposed to passive smoking were found to have higher levels of papillary gingival pigmentation compared to the children exposed to passive smoking.^28^

#### Smoking Exposure

The most common method to determine SHS exposure, and subsequent passive smoking, was through structured questionnaires. However, the definition for SHS and passive smoking varied between studies. In general, passive smoking was defined to include at least one parent smoking at least 20 cigarettes a day and where the smoking occurs indoors.^31^ Another study also included public spaces, in addition to smoking within the home, as part of the criteria for passive smoking.^36^

The majority of studies (81.8%) reported a strong correlation between prenatal smoking exposure and dental caries prevalence.^24,25,28–30,33,34,39,40^ The remaining two studies reported no association.^14,36^ A 40% increase in dental caries was found in children where mothers reported smoking during their first trimester of pregnancy.^33^ Similarly, four other cross-sectional studies investigating maternal prenatal smoking also found higher dmfs scores of children overall compared to ­children of non-smoking mothers during pregnancy.^28–30,33^ More than half the children with high dental caries levels from prenatal smoking exposure were associated with low-income families.^30^ A cohort study determined a link between ­prenatal smoking, low birth weight, and an increased prevalence of dental caries in children.^26^ Dental caries susceptibility was enhanced in children exposed to ­prenatal smoking compared to postnatal smoking in two cross-sectional ­studies.^35,43^

Postnatal passive smoking was reported in 20 studies of which 95% (*n* = 19) showed a positive exposure–­response relationship with passive smoking and dental ­caries.^5,11,12,14,16,23,26–28,30,32–38,41,42^ Children with family members who smoked had significantly more dental caries than those with non-smokers.^14^ Only one study found that paternal smoking in the household affected dental caries in both infants and children.^39^

All 12 studies which reported maternal smoking showed a positive association with passive smoking,^16,24–30,33,35,38,40^ whereas 19 of 21 studies (90.5%) found similar results for household smoking.^5,11,12,14,16,23,24,27,28,30,32–34,36–39,41,42^

#### Confounding Factors

Several confounding factors were reported from the included studies. Gender was considered in 20 studies (80%),^11,12,14,16,23–27,30–38,41,42^ but only two studies (8%) found an increase in dental caries in males compared to females.^11,12^

Poor dietary habits, including increased sugar intake and increased snacking frequency, were associated with increased prevalence of dental caries in six studies.^11,23,26,32,33,36^ The frequency of sugar intake and snacking was greater in households exposed to passive smoking was reported in seven studies.^12,16,24,25,28,30,34^

Parental education and family income, both indicators of SES, were investigated in 15^11,12,14,23–27,29,33,34,36,37,41,42^ and 10^11,14,23,26,29,32–34,37,41,42,44^ studies, respectively. Higher ­parental education was associated with a decrease in passive smoking and ultimately reduced dental caries experience.^11,12,23–27,29,33,34,41,42^ No significant differences associated with the level of parental education and dental caries exposure was reported in two studies.^36,37^ Lower health literacy of parents and care givers indicated their reluctance to believe the harmful effects of passive smoking and increased risk of continuing with household and car smoking habits around infants and children in one study.^42^

Increased SES of the household was associated with the decreased risk of dental caries.,^23,26,33^ whereas five studies found a higher risk of dental caries and passive smoking in children born in lower SES countries.^29,32,35,41,42^ Maternal smoking significantly decreased if both parents were employed, when compared to one, or neither, parent being employed.^11^ A positive association between passive smoking and dental caries remained after accounting for low SES. No significant differences between passive smoking and SES was reported in one study only.^37^

Mothers who smoke have poorer oral health behavior, including poor brushing frequency, and reduced fluoride use.^8,12,28,43^ Children exposed to environmental and household smoking versus those not exposed, showed behavior pattern differences resulting in poorer brushing and dietary habits, and a subsequent increased dental caries risk.^14^

After accounting for lower parental education, SES, dietary habits, oral hygiene, and gender as confounding factors, these studies found a positive association between SHS and dental caries risk.

## Discussion

Dental caries is the most widespread non-communicable disease across the world, significantly affecting health and well-being, quality of life, social interactions, rates of absenteeism from school or work, and posing a significant financial burden at both an individual and government levels.^45^ Dental caries results from the phasic demineralization and remineralization of tooth surfaces, with progression determined by several pathologic and protective factors. Fermentable carbohydrates on the tooth surface act as a substrate for acidogenic bacteria, which lower the salivary and plaque pH and initiate demineralization.^46^ Frequent exposure to sugars, poor oral hygiene, prevalence of high-risk acidogenic bacteria (*Streptococcus mutans*), and xerostomia are all pathological factors. Protective factors include fluoride, calcium, high salivary flow, and buffering capacity, and antibacterial agents.^47^ These were all considered as confounding factors in this study. In children, untreated dental caries can result in significant morbidity due to pain, difficulty in eating, speech impairment, premature deciduous tooth loss, damage to the permanent dentition, reduced self-esteem, and behavioral disturbances.^8,48,49^

The biological link between passive smoking and dental caries is plausible. Nicotine increases the proliferation of *S. mutans* directly, thus increasing the prevalence of this cariogenic bacterium in the mouths of children exposed to passive smoking.^50^ There is also strong evidence confirming that *S. mutans* are transmitted from mother to child during infancy.^51^ Thus, a child exposed to a smoking parent with a high bacterial load may be subject to higher transfer of bacteria, earlier exposure to *S. mutans*, and higher counts compared to a child with non-smoking parents.^15^ This theory is supported by a case–control study which showed greater mean *Streptococcus* and *Lactobacillus* counts in children from smoking households compared to controls.^52^ Passive smoking has also been shown to increase the risk of respiratory conditions and mouth breathing, leading to xerostomia.^53^ Consequently, the protective properties of saliva may be lost, including dilution and elimination of sugars, buffering capacity, calcium release, immunological, and bacteriostatic factors, creating a more favorable environment for dental caries.^15^ ETS may also decrease vitamin C, which increases acidogenic bacterial growth.^15^

The results of this systematic review strongly support the association between passive smoking and dental caries in the deciduous dentition of 0- to 6-year-old children. Over 94% of the included studies found that dental caries increased significantly after exposure to ETS (Table 2). A dose–response relationship between degree of passive smoking and dmft scores further supports the likelihood that such associations are related to tobacco exposure rather than other confounding factors.^54^

Compared to postnatal smoking alone, prenatal exposure to passive has been hypothesized to enhance susceptibility to dental caries. This systematic review found over 80% of studies supporting this association, with increased rates of dental caries experienced in children exposed to prenatal smoking (Table 2). A higher dental caries prevalence was reported in children with prenatal smoking exposure compared to postnatal maternal or household exposure.^35^ This suggests that dental hard tissues subjected to passive smoking are altered during development. Pregnant women who did not smoke themselves, but lived in a household where smoking occurred, gave birth to infants with significantly high cotinine levels, demonstrating that the harmful effects of smoking are easily transferred between mother and infant.^5^ Exposure to nicotine from cigarette smoking during pregnancy can also affect mineralization of enamel, allowing more dental biofilm (plaque) to be trapped on these surfaces, thereby contributing to increased dental caries risk.^16^

Hypo-mineralized teeth are more susceptible to dental caries.^55^ Prenatal exposure to passive smoking may therefore impact significantly on the mineralization and dental caries resistance of the developing dentition. Prenatal smoking has also been shown to cause premature tooth eruption,^44^ suggesting that these hypo-mineralized teeth are exposed to the oral environment for longer with a higher risk for dental caries development.

No specific correlations between passive smoking and gingival pigmentation or gingivitis were reported in any of the included articles. However, the biological link between passive smoking and periodontal disease (including gingivitis) has been well established in subjects older than six.^56^ Tobacco promotes invasion of *Porphyromonas gingivalis*, inhibits the immune response, and aggravates the inflammatory response, increasing the rate of periodontal ligament, and alveolar bone destruction.^57^ Nicotine also causes vasoconstriction, delaying wound healing and making gingivitis less clinically evident and more easily missed.^57^ Considering the strong associations in those older than six, the biological plausibility and possible risks, further research to assess this association in the 0- to 6-year-old group is encouraged.

A positive association between salivary cotinine levels and passive smoking was found in all seven studies where this was investigated. This confirms salivary cotinine as an accurate, reliable, and minimally invasive biomarker to determine the degree of passive smoking. It is recommended that any future studies on the effects of passive smoking includes salivary cotinine measurement. Salivary cotinine may also be used as part of pediatric screening programs to determine dental caries risk, allowing early intervention, parental education, and implementation of oral health programs to reduce dental caries development in these at-risk children.

Children growing up in disadvantaged and low SES backgrounds are subjected more to passive smoking.^58^ Low SES parents display a lack of attention to oral hygiene and routine dental appointments, thereby resulting in a higher risk for dental caries development.^14,59^ Parental oral health attitudes, especially in low SES households, directly affect children’s oral hygiene habits and dietary intake, thereby increasing prevalence of dental decay.^60^ Maternal postnatal smoking may also be an indication that mothers may indulge in risk-taking behaviors and poor dental habits, affecting the upbringing of their children.^26^

A limitation identified by this review was the lack of a standard definition for SHS and passive smoking, as well as only 13 of the 25 included studies (52%) presenting with a low risk of bias according to the NOS criteria.^11,27–34,37–39,42^ The diverse range of included studies, with varying methodologies and indices, may also have affected heterogeneity of data. Selection bias and associated publication bias were minimized by utilizing three different search engines, manual hand searching, as well as implementing a duplicate screening strategy by independent reviewers.

The majority of included studies were of a cross-sectional design (76%), which limits the assessment of causality for passive smoking and oral conditions. More prospective studies on this topic are encouraged to confirm causation of passive smoking on oral health. It should also be noted that considering the large number of protective and pathological factors associated with tooth development, mineralization, and both pre-and postnatal dental caries, it is difficult to fully account for confounding factors. After accounting for lower parental education, SES, dietary habits, oral hygiene, and gender as confounding factors, these studies still found a positive dose-response association between passive smoking and dental caries risk.

The majority of included studies only assessed passive smoking by use of a questionnaire, which could affect response bias.^6^ Salivary cotinine measurement is a reliable and valid method of measuring the exact degree of exposure to ETS and passive smoking should be included in all future studies to overcome response bias. This would allow determination of more accurate dose–response relationships, or percentage increase in dental caries risk for specific degree of exposure.

## Conclusions

This review provided an update on existing data by including more recent literature and studies examining the effects of passive smoking exposure on the oral health conditions of 0- to 6-year-old infants and children. In particular, the results of this systematic review strongly suggest a significant association between dental caries in the deciduous dentition and passive smoking. The evidence that ETS and passive smoking, prenatally, and postnatally during early childhood, is a risk factor for oral health conditions, justifies all health professionals paying more attention to passive smoking when conducting pediatric patient histories. Including salivary cotinine measurement in health questionnaires may assist with measuring the degree of passive smoking exposure in infants and children and allow for education and prevention in high-risk children. Early intervention and appropriate parental education regarding SHS effects on infants and children will allow for the minimization of dental caries, improvement in oral health outcomes, and overall reduction in smoking-associated systemic conditions for the children exposed. Thorough questionnaires, history taking, and examinations will contribute to improved diagnosis and guide appropriate treatment planning and follow-up schedules for parents and caregivers regarding infants and children exposed to ETS.

## Supplementary Material

A Contributorship Form detailing each author’s specific involvement with this content, as well as any supplementary data, are available online at https://academic.oup.com/ntr.

ntad093_suppl_Supplementary_Tables

## Data Availability

Since no datasets were generated or analysed during the current study, data sharing is not applicable.

## References

[1] World Health Organization. *Tobacco*. World Health Organization. https://www.who.int/news-room/fact-sheets/detail/tobacco#:~:text=Tobacco%20kills%20more%20than%208,%2D%20and%20middle%2Dincome%20countries. Accessed July 24, 2022.

[2] World Health Organization. *International consultation on environmental tobacco smoke and child health*. World Health Organization. https://apps.who.int/iris/handle/10665/65930. Accessed January 23, 2022.

[3] DiGiacomo SI , Jazayeri M-A, Barua RS, Ambrose JA. Environmental tobacco smoke and cardiovascular disease. Int J Environ Res Public Health. 2018;16(1):96. 30602668 10.3390/ijerph16010096 PMC6339042

[4] Zhong Y , Tang Q, Tan B, Huang R. Correlation between maternal smoking during pregnancy and dental caries in children: a systematic review and meta-analysis. Front Oral Health. 2021;2:673449. doi:10.3389/froh.2021.673449 35048017 PMC8757723

[5] Kubo S , Adachi K. Evaluation of infants’ exposure to environmental tobacco smoke using salivary cotinine measurements. Br J Midwifery. 2017;25(6):366–371.

[6] González-Valero L , Montiel-Company JM, Bellot-Arcís C, et al. Association between passive tobacco exposure and caries in children and adolescents. A systematic review and meta-analysis. PLoS One. 2018;13(8):e0202497. 30114212 10.1371/journal.pone.0202497 PMC6095572

[7] Hajifattahi F , Azarshab M, Haghgoo R, Lesan S. Evaluation of the relationship between passive smoking and oral pigmentation in children. J Dent (Tehran). 2010;7(3):119–123. 21998785 PMC3184756

[8] Aligne CA , Moss ME, Auinger P, Weitzman M. Association of pediatric dental caries with passive smoking. JAMA. 2003;289(10):1258–1264. 12633187 10.1001/jama.289.10.1258

[9] Avşar A , Darka O, Topaloğlu B, Bek Y. Association of passive smoking with caries and related salivary biomarkers in young children. Arch Oral Biol. 2008;53(10):969–974. 18672230 10.1016/j.archoralbio.2008.05.007

[10] Dearing BA , Katz RV, Weitzman M. Prenatal tobacco and postbirth second-hand smoke exposure and dental caries in children. Community Dent Oral Epidemiol. 2022;50(2):130–138. 33846993 10.1111/cdoe.12642

[11] Lee ZL , Gan WY, Lim PY, Hasan R, Lim SY. Associations of nutritional status, sugar and second-hand smoke exposure with dental caries among 3- to 6-year old Malaysian preschoolers: a cross-sectional study. BMC Oral Health. 2020;20(1):164. doi:10.1186/s12903-020-01152-0 32493338 PMC7268511

[12] Leroy R , Hoppenbrouwers K, Jara A, Declerck D. Parental smoking behavior and caries experience in preschool children. Community Dent Oral Epidemiol. 2008;36(3):249–257. 18474057 10.1111/j.1600-0528.2007.00393.x

[13] Shenkin JD , Broffitt B, Levy SM, Warren JJ. The association between environmental tobacco smoke and primary tooth caries. J Public Health Dent. 2004;64(3):184–186. 15341143 10.1111/j.1752-7325.2004.tb02750.x

[14] Tanaka S , Shinzawa M, Tokumasu H, et al. Secondhand smoke and incidence of dental caries in deciduous teeth among children in Japan: population based retrospective cohort study. BMJ. 2015;351:h5397. doi:10.1136/bmj.h5397 26489750 PMC4613892

[15] Vellappally S , Fiala Z, Šmejkalová J, Jacob V, Shriharsha P. Influence of tobacco use in dental caries development. Cent Eur J Public Health. 2007;15(3):116–121. 17958204 10.21101/cejph.a3431

[16] Hanioka T , Nakamura E, Ojima M, Tanaka K, Aoyama H. Dental caries in 3-year-old children and smoking status of parents. Paediatr Perinat Epidemiol. 2008;22(6):546–550. 19000292 10.1111/j.1365-3016.2008.00950.x

[17] Moher D , Liberati A, Tetzlaff J, Altman DG; PRISMA Group. Preferred reporting items for systematic reviews and meta-analyses: the PRISMA statement. PLoS Med. 2009;6(7):e1000097. 19621072 10.1371/journal.pmed.1000097 PMC2707599

[18] Morgan RL , Whaley P, Thayer KA, Schünemann HJ. Identifying the PECO: a framework for formulating good questions to explore the association of environmental and other exposures with health outcomes. Environ Int. 2018;121(Part 1):1027–1031. 30166065 10.1016/j.envint.2018.07.015 PMC6908441

[19] McHugh ML. Interrater reliability: the kappa statistic. Biochem Med. 2012;22(3):276–282. PMC3900052 23092060

[20] Wells GA , Shea B, O’Connell D, et al. *The Newcastle-Ottawa Scale (NOS) for assessing the quality of nonrandomised studies in meta-analyses* . Ottawa Hospital Research Institute. https://www.ohri.ca/programs/clinical_epidemiology/oxford.asp. Accessed January 23, 2022.

[21] Modesti PA , Reboldi G, Cappuccio FP, et al; ESH Working Group on CV Risk in Low Resource Settings. Panethnic differences in blood pressure in Europe: a systematic review and meta-analysis. PLoS One. 2016;11(1):e0147601. 26808317 10.1371/journal.pone.0147601 PMC4725677

[22] Higgins JPT , Thomas J, Chandler J, et al. *Cochrane Handbook for Systematic Reviews of Interventions version 6.3 (updated February 2022)* . Cochrane. https://training.cochrane.org/handbook/current. Accessed 23 April, 2022.

[23] Aida J , Ando Y, Oosaka M, Niimi K, Morita M. Contributions of social context to inequality in dental caries: a multilevel analysis of Japanese 3-year-old children. Community Dent Oral Epidemiol. 2008;36(2):149–156. 18333879 10.1111/j.1600-0528.2007.00380.x

[24] Akinkugbe AA. Cigarettes, e-cigarettes, and adolescents’ oral health: findings from the Population Assessment of Tobacco and Health (PATH) study. JDR Clin Transl Res. 2019;4(3):276–283. doi:10.1177/2380084418806870 30931714

[25] Akinkugbe AA. Does the trimester of smoking matter in the association between prenatal smoking and the risk of early childhood caries? Caries Res. 2021;55(2):114–121. 33508853 10.1159/000513257 PMC8035257

[26] Bernabé E , MacRitchie H, Longbottom C, Pitts NB, Sabbah W. Birth weight, breastfeeding, maternal smoking and caries trajectories. J Dent Res. 2017;96(2):171–178. 27834298 10.1177/0022034516678181

[27] Goto Y , Wada K, Konishi K, et al. Association between exposure to household smoking and dental caries in preschool children: a cross-sectional study. Environ Health Prev Med. 2019;24(1):9. doi:10.1186/s12199-019-0764-1 30684963 PMC6347787

[28] Hasmun NN , Drummond BK, Milne T, et al. Effects of environmental tobacco smoke on the oral health of preschool children. Eur Arch Paediatr Dent. 2017;18(6):393–398. 29090450 10.1007/s40368-017-0308-6

[29] Julihn A , Soares FC, Hjern A, Dahllöf G. Socioeconomic determinants, maternal health, and caries in young children. JDR Clin Transl Res. 2018;3(4):395–404. doi:10.1177/2380084418788066 PMC6139990 30263967

[30] Majorana A , Cagetti MG, Bardellini E, et al. Feeding and smoking habits as cumulative risk factors for early childhood caries in toddlers, after adjustment for several behavioral determinants: a retrospective study. BMC Pediatr. 2014;14:45. doi:10.1186/1471-2431-14-45 24528500 PMC3930287

[31] Mohammed AY , Hoobi NM. Dental caries experience and salivary total protein among 5 years passive smokers in Tikrit City, Iraq. Indian J Public Health Res Dev. 2019;10(11):1773–1777.

[32] Nakayama Y , Mori M. Association of environmental tobacco smoke and snacking habits with the risk of early childhood caries among 3-year-old Japanese children. J Public Health Dent. 2015;75(2):157–162. 25659226 10.1111/jphd.12085

[33] Tanaka K , Miyake Y, Nagata C, Furukawa S, Arakawa M. Association of prenatal exposure to maternal smoking and postnatal exposure to household smoking with dental caries in 3-year-old Japanese children. Environ Res. 2015;143(pt A):148–153. 26492399 10.1016/j.envres.2015.10.004

[34] Tanaka K , Miyake Y, Sasaki S. The effect of maternal smoking during pregnancy and postnatal household smoking on dental caries in young children. J Pediatr. 2009;155(3):410–415. 19555966 10.1016/j.jpeds.2009.03.032

[35] Williams SA , Kwan SYL, Parsons S. Parental smoking practices and caries experience in pre-school children. Caries Res. 2000;34(2):117–122. 10773628 10.1159/000016578

[36] Tang S-D , Zhang Y-X, Chen L-M, et al. Influence of life-style factors, including second-hand smoke, on dental caries among 3-year-old children in Wuxi, China. J Paediatr Child Health. 2020;56(2):231–236. 31408250 10.1111/jpc.14566

[37] Avşar A , Darka O, Bodrumlu EH, Bek Y. Evaluation of the relationship between passive smoking and salivary electrolytes, ­protein, ­secretory IgA, sialic acid and amylase in young children. Arch Oral Biol. 2009;54(5):457–463. 19249013 10.1016/j.archoralbio.2009.01.017

[38] Mills LM , Semple SE, Wilson IS, et al. Factors influencing exposure to secondhand smoke in preschool children living with smoking mothers. Nicotine Tob Res. 2012;14(12):1435–1444. 22422926 10.1093/ntr/nts074

[39] Sachiyo K , Kumiko A, Keiko N, Kaori K, Sonomi O. Effect of passive smoking using maternal and neonatal salivary cotinine measurements. Nurs Res. 2012;61(2):140–144. 22282157 10.1097/NNR.0b013e3182456690

[40] Sherif NA , Kamel SM, Al-Ashkar OS, et al. Detection of cotinine in neonate meconium as a marker for nicotine exposure in utero. East Mediterr Health J. 2004;10(1–2):96–105. 16201714

[41] Warren JR , Sloan P, Allen M, Okuyemi KS. Exploring children’s secondhand smoke exposure with early child care providers. Am J Prev Med. 2010;39(6 suppl 1):S44–S47. 21074677 10.1016/j.amepre.2010.09.005

[42] Welkom JS , Riekert KA, Rand CS, Eakin MN. Associations between caregiver health literacy and preschool children’s secondhand smoke exposure. J Pediatr Psychol. 2016;41(4):462–472. 26330535 10.1093/jpepsy/jsv077 PMC5009453

[43] Hanioka T , Ojima M, Tanaka K, Yamamoto M. Does secondhand smoke affect the development of dental caries in children? A systematic review. Int J Environ Res Public Health. 2011;8(5):1503–1519. 21655133 10.3390/ijerph8051503 PMC3108123

[44] Ntani G , Day PF, Baird J, et al; Southampton Women’s Survey Study Group. Maternal and early life factors of tooth emergence patterns and number of teeth at 1 and 2 years of age. J Dev Orig Health Dis. 2015;6(4):299–307. 25936832 10.1017/S2040174415001130 PMC4538790

[45] World Health Organization. Sugars and dental caries. World Health Organization. https://www.who.int/news-room/fact-sheets/detail/sugars-and-dental-caries. Accessed January 23, 2022.

[46] Touger-Decker R , Van Loveren C. Sugars and dental caries. Am J Clin Nutr. 2003;78(4):881S–892S. 14522753 10.1093/ajcn/78.4.881S

[47] Maheswari SU , Raja J, Kumar A, Seelan RG. Caries management by risk assessment: a review on current strategies for caries prevention and management. J Pharm Bioallied Sci. 2015;7(suppl 2):S320–S324. 26538870 10.4103/0975-7406.163436 PMC4606612

[48] Casamassimo PS , Thikkurissy S, Edelstein BL, Maiorini E. Beyond the dmft: the human and economic cost of early childhood caries. J Am Dent Assoc. 2009;140(6):650–657. 19491160 10.14219/jada.archive.2009.0250

[49] Kazeminia M , Abdi A, Shohaimi S, et al. Dental caries in primary and permanent teeth in children’s worldwide, 1995 to 2019: a systematic review and meta-analysis. Head Face Med. 2020;16(1):22. doi:10.1186/s13005-020-00237-z 33023617 PMC7541284

[50] Wu J , Li M, Huang R. The effect of smoking on caries-related microorganisms. Tob Induc Dis. 2019;17:32. doi:10.18332/tid/105913 31516475 PMC6662784

[51] Damle SG , Yadav R, Garg S, et al. Transmission of mutans streptococci in mother-child pairs. Indian J Med Res. 2016;144(2):264–270. 27934807 10.4103/0971-5916.195042 PMC5206879

[52] Menon I , Bhat N. Association of passive smoking with dental caries and salivary biomarkers among 5–10 years old children of Muradnagar, Ghaziabad. J Family Med Prim Care. 2019;8(8):2633–2639. 31548946 10.4103/jfmpc.jfmpc_369_19 PMC6753823

[53] Hur K , Liang J, Lin SY. The role of secondhand smoke in allergic rhinitis: a systematic review. Int Forum Allergy Rhinol 2014;4(2):110–116. doi:10.1002/alr.21246 24493468

[54] Akinkugbe AA , Brickhouse TH, Nascimento MM, Slade GD. Prenatal smoking and the risk of early childhood caries: a prospective cohort study. Prev Med Rep. 2020;20:101201. doi:10.1016/j.pmedr.2020.101201 33083206 PMC7554205

[55] Almuallem Z , Busuttil-Naudi A. Molar incisor hypomineralisation (MIH)—an overview. Br Dent J. 2018;225(7):601–609. 10.1038/sj.bdj.2018.814 30287963

[56] Akinkugbe AA , Slade GD, Divaris K, Poole C. Systematic review and meta-analysis of the association between exposure to environmental tobacco smoke and periodontitis endpoints among nonsmokers. Nicotine Tob Res. 2016;18(11):2047–2056. 27083214 10.1093/ntr/ntw105 PMC5055738

[57] Zhang Y , He J, He B, Huang R, Li M. Effect of tobacco on periodontal disease and oral cancer. Tob Induc Dis. 2019;17:40. doi:10.18332/tid/106187 31516483 PMC6662776

[58] Treyster Z , Gitterman B. Second hand smoke exposure in children: environmental factors, physiological effects, and interventions within pediatrics. Rev Environ Health. 2011;26(3):187–195. 22206195 10.1515/reveh.2011.026

[59] Chen L , Hong J, Xiong D, et al. Are parents’ education levels associated with either their oral health knowledge or their children’s oral health behaviors? A survey of 8446 families in Wuhan. BMC Oral Health. 2020;20(1):203. doi:10.1186/s12903-020-01186-4 32652985 PMC7353758

[60] Vanagas G , Milašauskienė Z, Grabauskas V, Mickevičienė A. Associations between parental skills and their attitudes toward importance to develop good oral hygiene skills in their children. Medicina. 2009;45(9):718–723. 19834309

