# Supplementary material for: Passive Smoking and Oral Health of Infants, Preschoolers, and Children: A Systematic Review
Source: Nicotine Tob Res. 2023 Jun 13;25(10):1625–32. doi: 10.1093/ntr/ntad093 (PMC10445258; doi:10.1093/ntr/ntad093)
Supplement: ntad093_suppl_Supplementary_Tables [file ntad093_suppl_supplementary_tables.docx]

**Supplementary Table S1.** Extraction of data

| Author  Year  Country | Age of participants | Sample size  (n) | Risk of bias | Direction of significance | | |  |
| --- | --- | --- | --- | --- | --- | --- | --- |
|  |  |  |  | Health variables | Smoking exposure | Confounding factors |  |
| Cohort studies (n=5) | | | | | | | |
| Akinkugbe et al. ^54^  2019  England | 0-5 years | 1,429 | High | Dental caries (+) | Prenatal (+)  Maternal (+)  Household (+) | Gender  Diet  Parental Education |  |
| Akinkugbe^25^  2021  England | 2.5-5 years | 1,429 | High | Dental caries (+) | Prenatal (+)  Maternal (+) | Gender  Diet  Parental education  Oral Hygiene  Fluoride exposure |  |
| Bernabé et al. ^26^  2017  Scotland | 1-4 years | 1,102 | High | Dental caries (+) | Postnatal (+)  Maternal (+) | Gender  Diet  Parental education  Family income  Oral Hygiene |  |
| Kubo et al. ^5^  2017  Japan | 1-10 months | 1 month - 71  10 months - 33 (progressive drop out) | High | Salivary Cotinine (+) | Postnatal (+)  Household (+) |  |  |
| Tanaka et al. ^33^  2015  Japan | 4 months- 3 years | 76,920 | Low | Dental caries (+) | Prenatal (0)  Postnatal (+)  Household (+) | Gender  Diet  Parental education  Family income  Oral Hygiene  Fluoride Exposure |  |
| Case control studies (n = 1) | | | | | | | |
| Tang et al. ^36^  2020  China | 3 years | 283 | High | Dental caries (0) | Prenatal (0)  Postnatal (+)  Household (+) | Gender  Diet  Parental education  Oral Hygiene  Fluoride exposure |  |
| Cross-sectional studies (n= 19) | | | | | | | |
| Aida et al. ^23^  2008  Japan | 3 years | 3,068 | Medium | Dental caries (+) | Postnatal (+)  Household (+) | Gender  Diet  Parental education  Family income  Oral Hygiene  Fluoride exposure |  |
| Avşar et al. ^37^  2009  Turkey | 4-6 years | 180 | Low | Salivary Cotinine (+) | Postnatal (+)  Household (+) | Gender  Diet  Parental education  Family income  Oral Hygiene |  |
| Hasmun et al. ^28^  2017  New Zealand | 1-5 years | 44 | Low | Dental caries (+)  Gingivitis (0)  Gingival Pigmentation (-) | Prenatal (+)  Postnatal (+)  Maternal (+)  Household (+) | Diet  Fluoride exposure |  |
| Goto et al. ^27^  2019  Japan | 3-6 years | 405 | Low | Dental caries (+) | Postnatal (+)  Maternal (+)  Household (+) | Gender  Diet  Parental education Oral Hygiene |  |
| Hanioka et al. ^16^  2008  Japan | 3 years | 711 | High | Dental caries (+) | Postnatal (+)  Maternal (+)  Household (+) | Gender  Diet  Oral Hygiene  Fluoride exposure |  |
| Julihn et al. ^29^  2018  Sweden | 3 years | 73,658 | Low | Dental caries (+) | Prenatal (+)  Maternal (+) | Parental education  Family income |  |
| Lee et al. ^11^  2020  Malaysia | 3-6 years | 396 | Low | Dental caries (+) | Postnatal (+)  Household (+) | Gender (male > female)  Diet  Parental education  Family income |  |
| Leroy et al. ^12^  2008  Belgium | 3 and 5 years | 2,533 | Medium | Dental caries (+) | Postnatal (+)  Household (+) | Gender (male > female)  Diet  Parental education  Oral Hygiene |  |
| Majorana et al. ^30^  2014  Italy | 2-2.5 years | 2,395 | Low | Dental caries (+) | Prenatal (+)  Postnatal (+)  Maternal (+)  Household (+) | Gender  Diet |  |
| Mills et al. ^38^  2012  Scotland | 1-5 years | 54 | Low | Salivary Cotinine (+) | Postnatal (+)  Maternal (+)  Household (+) | Gender |  |
| Mohammed et al. ^31^  2019  Iraq | 5 years | 60 | Low | Dental caries (+) | Postnatal (0)  Household (0) | Gender |  |
| Nakayama et al. ^32^  2015  Japan | 3 years | 1,801 | Low | Dental caries (+) | Postnatal (+)  Household (+) | Gender  Diet  Family income  Oral Hygiene  Fluoride exposure |  |
| Sachiyo et al. ^39^  2012  Japan | Neonates (within 24 hours of delivery) | 34 | Low | Salivary Cotinine (+) | Prenatal (+)  Household (+) |  |  |
| Sherif et al. ^40^  2004  Egypt | Neonates | 30 | Medium | Salivary Cotinine (+) | Prenatal (+)  Maternal (+) |  |  |
| Tanaka et al. ^34^  2009  Japan | 3 years | 2,015 | Low | Dental caries (+) | Prenatal (+)  Postnatal (+)  Household (+) | Gender  Diet  Parental education  Family income  Oral Hygiene  Fluoride exposure |  |
| Tanaka et al. ^14^  2015  Japan | 3 years | 6,412 | Medium | Dental caries (+) | Prenatal (+)  Postnatal (+)  Maternal (+)  Household (+) | Gender  Diet  Parental education  Family income  Oral Hygiene  Fluoride exposure |  |
| Warren et al. ^41^  2010  United States | 6 weeks – 5 years | 63 | High | Salivary Cotinine (+) | Postnatal (+)  Household (+) | Gender  Parental education  Family income |  |
| Welkom et al. ^42^  2015  United States | 6 months – 6 years | 268 | Low | Salivary Cotinine (+) | Postnatal (+)  Household (+) | Gender  Parental education  Family income |  |
| Williams et al. ^35^  2000  United Kingdom | 3-4.5 years | 763 | Medium | Dental caries (+) | Postnatal (+)  Maternal (+)  Household (0) | Gender  Diet  Social Class |  |

**Supplementary Table S2.** Risk of bias analysis

| Cohort studies (n=5) | | | | | | | | | |
| --- | --- | --- | --- | --- | --- | --- | --- | --- | --- |
| Author | Selection | | | | Comparability | | Outcome | | Total score  (Max: 9) (Risk) |
|  | Representa-tiveness of exposed cohort  (Max: *) | Selection of non-exposed cohort  (Max: *) | Ascertain-ment of exposure  (Max: *) | Demonstra-tion outcome not present at start of study  (Max: *) | Comparability of cohorts since design/analy-sis  (Max: **) | Assessment of outcome  (Max: *) | Follow-up long enough for outcomes to occur  (Max: *) | Follow-up adequacy of cohorts  (Max: *) |  |
| Akinkugbe et al. ^54^ | * | * |  |  | * | * | * |  | 5  (High) |
| Akinkugbe^25^ | * | * |  |  | * | * | * |  | 5  (High) |
| Bernabé et al. ^26^ | * | * |  |  | * |  | * | * | 5  (High) |
| Kubo et al. ^5^ |  |  |  |  |  | * | * | * | 3  (High) |
| Tanaka et al. ^33^ | * | * | * | * | * | * | * | * | 8  (Low) |
| Case control studies (n = 1) | | | | | | | | | |
| Author | Selection | | | | Comparability | Outcome | | | Total score  (Max: 9) (Risk) |
|  | Adequate case definition  (Max: *) | Representa-tiveness of the case  (Max: *) | Selection of controls  (Max: *) | Definition of controls  (Max: *) | Comparability of cases and controls on basis of the design/analy-sis  (Max: **) | Ascertain-ment of exposure  (Max: *) | Same method of ascertainment for cases and controls  (Max: *) | Non-response rate  (Max: *) |  |
| Tang et al. ^36^ |  |  |  |  |  |  |  |  | 0  (High) |
| Cross-sectional studies (n= 19) | | | | | | | | |  |
| Author | Selection | | | | Comparability | Outcome | | Total score  (Max: 10) (Risk) |  |
|  | Representat-iveness of sample  (Max: *) | Sample size  (Max: *) | Non-respondents  (Max: *) | Ascertain-ment of exposure  (Max: **) | Comparability of subjects on basis of design/analy-sis. Confounding factors are controlled  (Max: **) | Assessment of the outcome  (Max: **) | Statistical test  (Max: *) |  |  |
| Aida et al. ^23^ | * | * | * | * | * | * | * | 7  (Medium) |  |
| Avşar et al. ^37^ | * |  | * | ** | ** | ** | * | 9  (Low) |  |
| Hasmun et al. ^28^ | * | * | * | ** | * | ** | * | 9  (Low) |  |
| Goto et al. ^27^ | * | * | * | * | * | ** | * | 8  (Low) |  |
| Hanioka et al. ^16^ | * |  | * | * | * |  |  | 4  (High) |  |
| Julihn et al. ^29^ | * | * | * | ** | * | ** | * | 9  (Low) |  |
| Lee et al. ^11^ | * | * | * | ** | ** | ** | * | 10  (Low) |  |
| Leroy et al. ^12^ | * | * | * | * |  | ** | * | 7  (Medium) |  |
| Majorana et al. ^30^ | * | * | * | * | * | ** | * | 8  (Low) |  |
| Mills et al. ^38^ | * | * |  | ** |  | ** | * | 9  (Low) |  |
| Mohammed et al. ^31^ | * | * | * | ** | * | ** | * | 9  (Low) |  |
| Nakayama et al. ^32^ | * | * | * | ** | ** | ** | * | 10  (Low) |  |
| Sachiyo et al. ^39^ | * |  | * | ** | * | ** | * | 8  (Low) |  |
| Sherif et al. ^40^ | * |  |  | ** |  | ** | * | 6  (Medium) |  |
| Tanaka et al. ^34^ | * | * | * | * | * | ** | * | 8  (Low) |  |
| Tanaka et al. ^14^ | * | * | * | * |  | ** | * | 7  (Medium) |  |
| Warren et al. ^41^ |  |  |  |  |  |  |  | 0  (High) |  |
| Welkom et al. ^42^ | * | * | * | ** | * | ** | * | 9  (Low) |  |
| Williams et al. ^35^ | * | * | * | * | * | * | * | 7  (Medium) |  |
